# Adopting strategies with menopausal experiences: A systematic review

**DOI:** 10.1002/hsr2.1968

**Published:** 2024-04-17

**Authors:** Mansooreh Khandehroo, Nooshin Peyman, Mehrossadet Mahdizadeh, Maryam Salary, Hadi Tehrani

**Affiliations:** ^1^ Department of Health Education and Health Promotion, Faculty of Health Mashhad University of Medical Sciences Mashhad Iran; ^2^ Social Determinants of Health Research Center Mashhad University of Medical Sciences Mashhad Iran; ^3^ Department of biostatistics, Social Determinants of Health Research Center, Faculty of Health Mashhad University of Medical Sciences Mashhad Iran

**Keywords:** adopting, complication, cultural, menopause, social support

## Abstract

**Background and Aims:**

Menopause is one of the most significant stages in women's life. It is accompanied by many complications and a serious challenge. This study aimed to assess the menopause experiences of Iranian women and compatibility strategies.

**Methods:**

We searched PubMed, Web of Science (ISI), Scopus, Ovid, and the Iranian Clinical Trial Registry and Magiran, SID, from January 1990 to January 2021.

**Results:**

Psychological effects, sexual disorders, physical problems, bone pain, insomnia, fatigue, and hot flashes are all menopause experiences. Cultural factors, lifestyle, social factors, education level, employment and economic status, marital status, and the number of pregnancies and births can influence this experience. It is important that menopausal women are aware how menopausal compatibility and prepare for this period. Many factors have affected menopausal adopting strategies. Negative emotions, negative attitudes, worry, and anxiety, and their psychological effects exacerbate the annoying experiences of menopause and decelerate menopausal adoption.

**Conclusions:**

Social support and educational intervention were the practical menopausal adopting strategies. It will guarantee the health of menopausal women in the last third of their lives.

## 1 INTRODUCTION

Due to the increasing trend of postmenopausal women, menopausal health will be one of the most fundamental issues in societies.^1^, ^2^ The age of menopause is between 50 and 51 years in developed countries. The age of menopause in Iran is 46.8 ± 7.8 years.^3^ The mean age of menopause in Iran is lower than in the world, which can lead to many problems.

Menopausal experiences are different in scopes and intensities that affect women's interpersonal communication, society, family, and overall quality of life.^2^, ^3^ It can lead to many complications, such as emotional and social complications.^4^ So, menopause is named a biological, social, cultural, and emotional process.^5^

Favorable socioeconomic conditions are associated with better‐incoming jobs and higher education in couples that could accelerate compatibility with menopausal changes and senility. Menopause is close to old age or sometimes with it. It can have a substantial impact on women's health. In addition, some socio‐demographic variables and perceived health affect the severity of menopausal experiences.^6^

This disaster may last for many years. It may be uncomfortable and put pressure on the health system's budget. We live in a world today. Where we need individual and social flexibility and adaptability more than ever. These are due to many changes and challenges caused by technology and modern life. Adaptability and flexibility have played a significant role in health promotion. Adaptability helps to increase the ability to control impulses, emotions, or attitudes. If a person doesn't communicate with others and his social environment or adaptation is disrupted, behavioral disorders will appear. Compatible people can communicate better with their surrounding environment and overcome environmental changes. Adaptable people cope with environmental conditions and changes and exploit them to their advantage. Significantly, people adapt according to the new conditions. The complications of menopause have been explained in various studies, but there have been few solutions for compatibility with these changes. Many studies describe menopausal experiences. Some experimental, semi‐experimental, and descriptive studies have assessed women's reactions to these experiences. Women's compatibility is more important than the description of experiences. Adopting can substantially affect the intensity and quality of the experiences and improve the menopausal quality of life.

Many studies have mentioned menopausal experiences. However, the strategy to adapt to menopause has been explained in few interventional studies. This research made a summary and conclusion of these cases. Compatibility to this period of life, which includes about one third of women's lives, is a fundamental issue.^7^, ^8^ This systematic review can be practical for expressing the experiences of menopause and how compatibility with it. It could help manage menopausal experiences. So, this study was conducted to investigate strategies for adopting menopausal experiences. The research question is “What strategy do menopausal women use for coping with menopausal experiences?”

## 2 METHODS

### 2.1 Study design

We searched and assessed International electronic bibliographic databases from January 1990 to January 2020, including PubMed, Web of Science (ISI), Scopus and Ovid, and English and Persian languages that were done in Iran. This review was performed by the PRISMA (Preferred Reporting Items for Systematic Reviews and Meta‐Analyses) protocol. The research question was: What are the menopause experiences and adaptation strategies of Iranian women?

### 2.2 Inclusion and exclusion criteria

The inclusion criteria were as follows: (1) articles published in Persian and English; (2) data from a cross‐sectional, case‐control, experimental, and semi‐experimental phenomenological study of postmenopausal women in Iran between January 1990 and January 2020. The exclusion criteria were as follows: (1) editorials, speeches, comments, and conference abstracts; (2) insufficient characterization; (3) the lack of full text and necessary information; and (4) to avoid multiple publication biases in our study, duplicate publications were removed from the analysis.

### 2.3 Search strategy

In this research, we manage to investigate annoying menopausal experiences and compatibility strategies of Iranian women. The keywords, as shown in Table 1, are menopause, compatibility, cultural, social support and complication, sign, symptom, and Iran. We screened the title and abstract to determine the relevant papers. If there were doubts in the abstract, the full text was evaluated. The protocol of this study was recorded in PROSPERO (record number: 281264).

**Table 1:** Combination of words used to search articles.

(“Culture” [Mesh]) AND ((“Menopause” [Mesh]) OR “Post menopause” [Mesh])
(“Social Support” [Mesh]) AND ((“Menopause” [Mesh]) OR “Post menopause” [Mesh])
(“Economics” [Mesh]) AND ((“Menopause” [Mesh]) OR “Post menopause” [Mesh])
(“compatibility” [Mesh]) AND ((“Menopause” [Mesh]) OR “Post menopause” [Mesh])
(“sign” [Mesh]) AND ((“Menopause” [Mesh]) OR “Post menopause” [Mesh])
(“symptoms” [Mesh]) AND ((“Menopause” [Mesh]) OR “Post menopause” [Mesh])
(“complication” [Mesh]) AND ((“Menopause” [Mesh]) OR “Post menopause” [Mesh])
(“Culture” [Mesh]) OR “Social Support” [Mesh]) OR “Economics” [Mesh]) OR ((“compatibility” [Mesh]) OR “sign” [Mesh])) OR “symptom” [Mesh]) OR “complication” [Mesh])

### 2.4 Study selection

**Figure 1:**
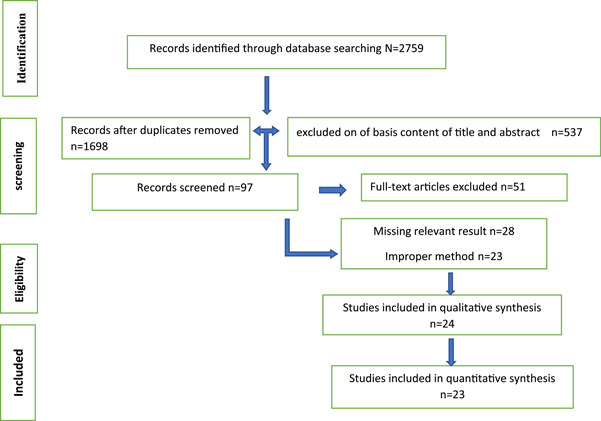
Flowchart of articles in systematic review.

We imported all the articles searched into the EndNote library. First, we provided titles and abstracts and further evaluated them. Then, we evaluated the full text of the article according to our inclusion and exclusion criteria. In this study, the eligible were reported, and the other noncompliant reports were deleted for the evidence shown in Figure 1.

### 2.5 Quality assessment

We downloaded all the potentially relevant studies from each database. Titles and abstracts of studies were assessed by two authors. Then, duplicate articles and those that did not fit with the study objective. Next, evaluate the Results and Methods section of the full text. So, the suitability of the remaining studies was evaluated by following exclusion and inclusion criteria, and non‐applicable or unrelated studies were deleted. Furthermore, the disagreement regarding the assessment results was resolved through a conversation with a third author. The quality of the articles was assessed by validated tools. The consort tool in clinical trials, the STORB tool (Strengthening the Reporting of Observation Studies in Epidemiology for observational research) for observational research, and the PRISMA tool for systematic review were used.

### 2.6 Data extraction

Data from the quantitative and qualitative studies were evaluated based on the objective of extracting data. Collected data onThe first author, title, type of study, year of publication, target group, sample size, questionnaire, type of intervention, and final impact of outcomes are written in Table 2.

**Table 2:** Data extracted from selected final articles.

	Author	Title	Type	Year	Sample	Size	Questionnaire	Intervention	Impact	Results
1	Nikpoure	Survey young women's knowledge of menopause	Descriptive	1891	45–65 years old	Indistinctive	Researcher made	‐	Determining knowledge	66% had low knowledge
2	Abrahimian	The effect of exercise on menopausal age	Case–control	2002	Menopausal women	90	Researcher made	‐	Determining effect of exercise on menopausal age	Exercise is effective in delaying menopausal age who have regular exercise
3	Arman	Comparison of sexual disorders in women before and after menopause	Cross‐sectional	2005	Menopausal women	174	Researcher made	‐	Survey	Sexual dysfunction increases with menopause
4	Golian	Promoting women's health at menopause	Hemi‐experimental	2007	Menopausal women	81	Researcher made	Education	Improvement knowledge	Education increased knowledge
5	Gamshidy manesh	Women's experience of menopause	Qualitative	2009	Menopausal women	14	Researcher made	‐	Survey	The most important themes are physiological and deprivation
6	Bahri	The relationship between the severity of menopausal symptoms and depression and anxiety in postmenopausal women	Cross‐sectional	2011	45–60 years old	100	Eshpringer questionnaire and cuperman questionnaire	Investigating	There isn't relationship between menopausal symptoms and depression and anxiety	There was a significant difference between the mean score of anxiety and the sign of “insomnia” in postmenopausal women. Severity of menopausal symptoms related to depression and anxiety
7	Rymaze	Female perceptions of menopausal phenomena in Iranian cultural context	Content analyze	2013	Menopausal women		Researcher made	Investigating	Investigating the perception of menopause	It is the end of women's sexual life
8	Moshky	Applying behavioral analysis in order investigate women's psychological well‐being During menopause	Cross‐sectional	2015	Menopausal women	110	Standard psychological well‐being questionnaire Ryff General Self‐efficacy questionnaire Sherer	Investigating	Increasing the level of knowledge and create a positive attitude and promoting healthy behaviors	A positive and significant correlation between predisposing factors and strengthening factors of psychological well‐being with menopause experiences
9	Shariat	Menopausal women's to social support	Descriptive	2015	Menopausal women	220	MSPSS perceived social support and assess the experiences of women in menopause questionnaires	Investigating	Family support as the wife is the best predictors of menopausal experiences	There are a significant correlation between social support and family support in the women experiences
10	Peyman and Khandehroo	Explaining women's perception of menopause	Phenomenology	2016	Menopausal women	36	‐	Investigating	Effective adaptation against menopause is an inevitable process of life	The main theme was menopause as an inevitable process of life
11	Bahri	Various treatments of menopause and its related factors	Cross‐sectional	2016	Menopausal women	460	Demographic and the treatment of menopausal symptoms questionnaires	Investigating	The most treatments are hormone therapy	The highest, 59.5% used hormone therapy and the lowest, 2.9% used acupuncture
12	Hakimy	Women's experiences of menopause	Descriptive	2017	Menopausal women	350	Demographic and Women's experience of menopause questionnaires	Investigating	The only predictor variable for women's menopausal experiences is the duration of menopause	Menopausal women's experiences included physical effects, negative emotions, negative attitudes, adjustment, worries and psychological effects
13	Hossein zadeh	Determining the validity and reliability of the symptom severity questionnaire Menopause MSSI‐38	Cross‐sectional	2018	Menopausal women	676	The symptom severity questionnaire Menopause MSSI‐38	Investigating	The tool is valid and reliable	CVI, CVR, AVE, CR determined
14	Serayeloo	Critical evaluation of published trials on the effect of complementary medicine on menopausal symptoms	Systematic review	2018	Articles	47	CONSORT check list	Review	Articles were mediocre	Calculation of sample size, blinding method, and the method used for the allocation sequence were not observed in almost all cases
15	Khavandy zadeh	The effect of education on the lifestyle of postmenopausal women	Semiexperimental	2018	Menopausal women	57	Unknown	Education	Positive effects of training on eating habits, exercise and walking, sun exposure, sleep and rest	The bedtime hours of the subjects were approximately 5 h before training and approximately 6 h after training
16	Gavady valla	Sexual arousal during the menopausal transition among Iranian women	Content	2018	Menopausal women	22	‐	Investigation	Decreased sexual capacity	Intimacy, socio‐cultural coding, a sense of youth
17	Morovatty	The effect of educational interventions based on the multitheory model on the quality of life of postmenopausal women	Cross‐sectional	2019	Menopausal women	24	MENQOL questionnaire	Education	Preparation of educational protocol	Education is effective in improving the quality of life after menopause
18	Yazdkhasty	Improving the menopause management model in Iranian women	Granded theory	2019	Menopausal women	30	Interview	Investigation	Improve menopausal management	The cultural context of menopause in Iran is a two‐pronged coin in which the threat to female identity is more than an opportunity
19	Heidary	Comparison of quality of life of urban and rural postmenopausal women	Cross‐sectional	2019	Menopausal women	312 urban 68 rural	Leiden‐Padua questionnaire	Investigation	Improve the quality of menopausal life	There was no significant difference in quality of life in urban and rural areas
20	Salimy Moghadam	The effect of group counseling based on GATHER approach on the quality of life of postmenopausal women with surgery	Clinical trial	2019	Menopausal women	78		Group counseling	Improve the quality of menopausal life	Improve the quality of menopausal life was seen in the test group
21	Namazy	Social determinants of health in menopause	Systematic review	2019	Menopausal women	40	Articles	Investigation	Determining social needs	Cultural factors, lifestyle (nutrition, exercise, smoking, etc.), family support, education level, are among the social factors that determine health
22	Nazar pour	Factors related to postmenopausal quality of life in Iranian women	Cross‐sectional	2020	Menopausal women	405	WHO Quality of Life‐BREF (WHOQOL‐BREF), the Menopause Rating Scale (MRS), and a researcher‐designed questionnaire	Investigation	Improve the quality of life	Social and cultural factors are closely related to menopausal symptoms that affect quality of life
23	Khandehroo	Health literacy intervention and quality of life in menopausal women: a randomized controlled trial	Clinical trial	2020	Menopausal women	60	(S‐TOFHLA), and (MENQOL) questionnaires	Training	Improve the quality of life	Increasing health literacy improved the quality of life in the experimental group

### 2.7 Data analysis

The database search results were 2759 articles. Titles and abstracts were evaluated after removing duplicate articles (*N* = 1698). In the next step, 537 articles had not been explained data correctly. Subsequently, the full text was evaluated. Twenty‐nine articles had uncompleted data. Twenty‐three articles had unclear methods. One of the articles had Poor quality. Finally, 23 articles were assessed by two authors. The disagreement on the evaluation results was resolved through a conversation with the third author. As shown in Figure 1, the flowchart is the description of the article selection process, based on the PRISMA checklist.

## 3 RESULTS

In total, 2759 articles were obtained through database research. Finally, 23 studies were selected for evaluation. Methodology of research in 23 selected articles was descriptive (*n* = 5, 21/7%) and cross‐sectional (*n* = 7, 30.4%), clinical trial (*n* = 3, 13.04%), semi‐experimental (*n* = 2, 8.6%), and qualitative (*n* = 5, 21.7%) included content analysis (*n* = 2, 8.6%), phenomenology (*n* = 2, 8.6%), and systematic review (*n* = 1, 4.3%). 3832 people participated in these articles. According to the studies, the menopausal age was 46/8 ± 7/8 years.^3^ Topics searched in these articles include: attitude^4^, ^9^, ^10^ (*n* = 3, 13.04%), experiences of menopause,^11^, ^12^, ^13^ (*n* = 3, 13.04%), social support,^6^, ^10^ (*n* = 2, 8.6%), psychological effects,^2^, ^13^, ^14^ (*n* = 3, 13.04%), sexual disorder,^15^, ^16^ (*n* = 2, 8.6%), quality of life.^7^, ^17^, ^18^, ^19^, ^20^, ^21^ (*n* = 6, 26.08%), and lifestyle^6^, ^21^, ^22^, ^23^ (*n* = 4, 17.3%).

### 3.1 Attitude

According to this review, attitudes toward menopause are influenced by the beliefs and culture of society.^4^, ^11^ Attitudes may include satisfaction or dissatisfaction.^4^ If menopause is a sign of aging, loss of youth, and loss of sexual attractiveness, this will lead to a negative attitude toward menopause.^1^, ^4^, ^12^, ^26^ In cultures where having children is the most important role for a woman, menopause creates a negative attitude in women.^4^, ^13^ In some societies and cultures, menopause gives women the freedom to have more opportunities to communicate with God and perform religious ceremonies, thus leading to a positive attitude among post‐menopausal women.^4^ Positive or negative attitudes toward menopause are one of the factors that affect the menopausal experiences.^1^, ^4^, ^11^, ^12^, ^13^

### 3.2 Somatic experiences

According to this review, most menopausal women had somatic experiences, the most common symptom of which was hot flashes.^24^ Hot flashes had been aggravated by stress and anxiety. As the age of postmenopausal women increased, the severity of physical and psychological symptoms decreased.^10^, ^25^ Increasing the level of education increases self‐confidence and better perception of health, thus improving the health of postmenopausal women and decreasing annoying Somatic experiences.^24^, ^26^, ^27^

### 3.3 Psychological experiences

According to this review, depression, anxiety and feeling lonely, irritability, impatience, and feelings of failure were psychological experiences during menopause.^13^ Psychological support from the husband, the most important person, decreased anxiety and depression and improved compatibility with menopause.^4^, ^6^ Marital satisfaction has reduced the psychological effects of menopause.^1^, ^4^, ^6^, ^13^ Various changes in the lives of post‐menopausal women, such as variations in the roles and responsibilities, have resulted in increased demands and expectations from myself and others. The changes in this had induced psychological dysfunctions.^13^, ^21^

### 3.4 Sexual experiences

According to this review, sexual capacity diminishes in the menopause.^13^ Many women have experienced complications in their sexual life during the transition from menopause.^13^, ^14^, ^15^, ^16^ Menopause had caused a great reduction or termination of sexual activity, but most women had considered themselves obligated to satisfy the sexual needs of their husbands.^13^, ^14^

### 3.5 Quality of life

According to this review, menopausal Changes had a significant impact on the quality of life and daily activities.^7^, ^18^, ^25^, ^28^ Quality of life decreases with aging.^7^ Educational interventions have reduced the complications of menopause, and these have improved the quality of life.^4^, ^18^, ^28^

### 3.6 Social support

According to the articles, social support was the level of love, help, and attention, from family members, friends, and others associated with the individual. Stress aggravated women's dependence on the family during menopause. Social and personality variables impacted the complications of menopause and health.^11^, ^12^ Social support has increased the ability of individuals to compatibility with stress and has reduced physical and psychological symptoms.^6^

### 3.7 Life style

According to this review, Exercise, physical activity, nonsmoking, and proper nutrition had been factored in menopausal health promotion,^11^, ^24^, ^28^ but aging has made it difficult to do physical activity and other things. Education has been one of the important factors in increased knowledge and improved performance of postmenopausal women for achieving health promotion.^7^

### 3.8 Adopting

According to this review, compatibility had been created by increasing the ability to control impulses and emotions.^4^ Women's view had determined their personal needs in menopausal compatibility. Useful entertainment also made women compatible with menopause. Many women mentioned entering the community as a useful factor and a way to prevent depression. Thus, they had been better compatible with menopause.^11^ Sometimes, the lack of knowledge about menopause could cause nonacceptance and the emergence of psycho‐emotional symptoms in post‐menopause.^7^ The educational intervention had accelerated compatibility with menopausal changes.^7^ The results of the research showed that behavioral intervention had reduced vasomotor symptoms in postmenopausal women.^4^, ^7^, ^14^, ^15^, ^24^, ^29^

## 4 DISCUSSION

This study showed that social support and educational intervention were the best compatibility strategies and improvement attitudes. Social support affects the experiences of menopause and has a significant role in reducing menopausal problems.^30^ Social activity compensates for the loss of fertility and decreases menopausal complications. As a result, working women experience fewer annoying experiences, complications, and problems. Communities can increase their social support by creating compatibilities for menopausal women to do charity work.

However, achieving optimal menopausal health requires considering many factors. Many factors affect postmenopausal psychological status, including psychological disorders, anxiety and depression, insomnia, and sexual dysfunction.^28^

Of course, some of these symptoms usually appear with aging; these symptoms are not related to menopause.^8^ Still, all of the factors would affect menopausal health. The review of these studies showed that psychological factors are one of the experiences affecting menopausal quality of life.

In one study, anxiety and depression did not correlate with the severity of menopausal Signs and symptoms in Iran,^10^ despite the research of other countries that have reported this correlation.^21^, ^23^, ^30^, ^31^ These differences are caused by differentiation in women's culture and attitudes. This attitude influences the experiences, signs, and symptoms or complications of menopause.

In some societies and cultures, such as Iran, menopause gives women liberate to have more opportunities to communicate with God and perform religious ceremonies, thus leading to a positive attitude in postmenopausal women.^4^

The thought that menopause is the end of sexual life leads to a negative attitude toward menopause.^15^ Still, “rescue feeling” or “liberation” from unwanted pregnancies, annoying dysmenorrhea, extensive bleeding, the negative reaction of sexual partners, and the health requirements of menstrual cycles lead to a positive attitude toward menopause.^4^, ^20^ A negative attitude toward menopause has a negative impact on menopausal adoption.^32^

Menopause can cause or increase sexual dysfunction. The prevalence of sexual disorders is 38% in the reproductive period and 72.4% in the postmenopausal period.^31^ Sexual disorders cause widespread family disputes and sometimes lead to family breakdowns. Using Royal jelly could be beneficial for vaginal dryness, and could be decreased sexual disorders, and improves menopausal health. Honey bee products Royal jelly, contains proteins, amino acids, sugars, fats, minerals, vitamins, gamma globulin, and elements.^33^ It influences the collagen production process and activates fibroblasts in the skin. Using it improves menopausal skin changes and sagging skin. It improves menopausal experiences and mental image in postmenopausal women.

Negative attitudes and aging feelings decrease if women prepare themselves for this phenomenon before menopause.^20^

The study of women's menopausal compatibility in England showed that 68% of women of psychological adjustment techniques, 66% of direct action, 63% of seeking social support, 58% of relaxation, 55% of redefining the situation, 48% of acceptance, and 36% of religion used to compatible menopause.^17^ According to research conducted in America, women who used task‐based coping strategies had better physical and mental health, compared to women who used emotional coping strategies.^6^ Similar results in 31 premenopausal and 36 post‐menopausal studies in Spain also revealed that individuals who used coping strategies instead of avoidance strategies compatible better with life changes caused by menopause.^18^

According to this review, education has a huge impact on menopausal compatibility and decreases annoying experiences,^6^, ^7^, ^20^, ^22^, ^24^, ^25^, ^32^ and improves menopausal health.^4^, ^10^ Rotem et al. demonstrated that giving efficient and updated information to postmenopausal women may improve their compatibility and ability to adapt menopause.^34^ They suggested that support group training programs have important effects on accepting physical, social, and psychological changes of menopause.^34^

Further studies should be performed on compatibility with menopause to identify healthy ways to deal with these problems. Education about these strategies can improve menopause compatibility. In this research, we tried to increase the knowledge of compatibility with menopause so that women can adapt to menopause more easily and have less annoying experiences.

### 4.1 Conclusion

The findings of this study show the importance of various factors in adapting to menopause. A positive attitude, increasing the support of husband, children, and peers, improving lifestyle, increasing awareness in this field, and participating in social interactions can help to adapt to menopause. These findings can be used in the formulation of a treatment‐care policy for menopausal women. It is suggested that future studies focus on how to create a positive attitude toward menopause, increase women's capabilities, and increase social support for menopausal women to maximize their adaptation to new conditions.

### 4.2 Limitations

One of the limitations is the small number of menopause compatibility studies and the unpublished results of other studies.

## AUTHOR CONTRIBUTIONS

**Mansooreh Khandehroo**: Conceptualization; data curation; investigation; methodology; project administration; validation; visualization; writing—original draft; writing—review and editing. **Nooshin Peyman**: Project administration; supervision. **Mehrossadet Mahdizadeh**: Formal analysis; methodology; writing—review and editing. **Maryam Salary**: Methodology; visualization. **Hadi Tehrani**: Methodology; supervision; validation.

## CONFLICT OF INTEREST STATEMENT

The authors declare no conflict of interest.

## ETHICS STATEMENT

All procedures performed in studies were by the ethical standards of the institutional research committee with the 1964 Helsinki Declaration. This proposal has been approved by the Ethics Committee of Mashhad University of Medical Sciences; the ethics code IR.MUMS.FHMPM.REC.1400.076. Address: https://ethics.research.ac.ir/IR.MUMS.FHMPM.REC.1400.076.

## TRANSPARENCY STATEMENT

The lead author Mansooreh Khandehroo, Nooshin Peyman affirms that this manuscript is an honest, accurate, and transparent account of the study being reported; that no important aspects of the study have been omitted; and that any discrepancies from the study as planned (and, if relevant, registered) have been explained.

## Data Availability

All data generated or analyzed during this study is included in this published article. All authors have read and approved the final version of the manuscript [CORRESPONDING AUTHOR or MANUSCRIPT GUARANTOR] had full access to all of the data in this study and took complete responsibility for the integrity of the data and the accuracy of the data analysis.
